# Transport of Amino Acids Across the Blood-Brain Barrier

**DOI:** 10.3389/fphys.2020.00973

**Published:** 2020-09-23

**Authors:** Rosa Zaragozá

**Affiliations:** Department of Human Anatomy and Embriology, School of Medicine, IIS INCLIVA, University of Valencia, Valencia, Spain

**Keywords:** blood-brain barrier, amino acid transport, facilitative transport, active transport, cell polarity, luminal membrane, abluminal membrane, endothelial cells

## Abstract

The blood-brain-barrier (BBB), present in brain capillaries, constitutes an essential barrier mechanism for normal functioning and development of the brain. The presence of tight junctions between adjacent endothelial cells restricts permeability and movement of molecules between extracellular fluid and plasma. The protein complexes that control cell-cell attachment also polarize cellular membrane, so that it can be divided into luminal (blood-facing) and abluminal (brain) sides, and each solute that enters/leaves the brain must cross both membranes. Several amino acid (AA) transport systems with different distributions on both sides of the BBB have been described. In a broad sense, there are at least five different systems of facilitative transporters and all of them are found in the luminal membrane. Some of these transporters are very specific for a small group of substrates and are located exclusively on the luminal side of the BBB. However, the two major facilitative carriers, system L and system y^+^, are located in both membranes, although asymmetrically. The position of these Na^+^-independent transporters ensures AA availability in the brain and also its bidirectional transport across the endothelial cells. On the other hand, there are several Na^+^-dependent transport systems that transport AAs against its concentration gradient together with the movement of Na^+^ ions. The majority of these active transporters are present exclusively at the abluminal membrane and are responsible for AA efflux from the brain into the endothelial cells. Since they are Na^+^-coupled, the sodium pump Na^+^/K^+^-ATPase is also highly expressed on this abluminal side of the BBB. Once inside the cell, the facilitative transporters located in the luminal membranes mediate export from the endothelial cell to the blood. In summary, the polarized distribution of these transport systems between the luminal and abluminal membranes, and the fact that more than one transporter may carry the same substrate, ensures supply and excretion of AAs in and out of the brain, thereby controlling its homeostasis and proper function.

## Introduction

### The Blood Brain Barrier

The term blood–brain barrier (BBB) was used to describe the unique characteristics of the microvasculature of the central nervous system (CNS). Such vessels within the CNS are continuous and non-fenestrated capillaries; moreover, endothelial cells (ECs) within the BBB are held together by continuous interendothelial tight junctions that limit paracellular transport across the endothelium (Brightman and Reese, 1969; Zlokovic, 2008). In fact, tight junctions of ECs give rise to a polarized cell composed of two distinct luminal (blood-side) and abluminal (brain-side) surfaces. Each of these membranes is made up of different lipids and intrinsic proteins (e.g., transporters) existing on the luminal and abluminal sides, that regulate movement of molecules between the blood and the brain (Van Meer and Simons, 1986; Tewes and Galla, 2001; Hawkins et al., 2006, 2013).

Nevertheless, endothelial cells forming the BBB are not the only mediators of this function. Regulation of this process does not only involve the EC tight junctions. There are mural cells surrounding the ECs that form the walls of capillaries. These mural cells, located on the abluminal side of the EC layer, include vascular smooth muscle cells around the large vessels, astrocytes and pericytes, which are embedded in the basement membrane and cover the abluminal side of the endothelial wall with their processes (Sims, 1986; Daneman and Prat, 2015). Several pericyte-endothelial cellular adhesions have been identified and due to the presence of contractile proteins within pericytes, these cells may control capillary diameter and thus blood flow (Peppiatt et al., 2006; Hall et al., 2014). In fact, these cells have been shown to be important for regulating the formation of the BBB during development, as well as maintaining its function in adulthood and aging (Daneman and Prat, 2015). Astrocytes are a major glial cell type, and their end-feet processes surround blood vessels and establish a neurovascular link, regulating blood flow in response to neuronal activity (Attwell et al., 2010).

The basement membrane, composed of collagen type IV, laminin, fibronectin and heparan sulfate proteoglycan, separates ECs from other cellular members interacting with them. These interactions are essential for barrier formation and maturation during development and are maintained thereafter. However, barrier function is not solely regulated by ECs, pericytes, astrocyte-processes and basement membrane. Neurons, microglial cells and perivascular macrophages also influence the BBB and thus a more extended term, neurovascular unit, was coined to include all these elements (Abbott et al., 2006; Neuwelt et al., 2011). The relationships between different cell types allow for paracrine modulation which, in turn, regulates CNS homeostasis, such as BBB permeability and blood flow (Zlokovic, 2008; Attwell et al., 2010).

### Transport Across the BBB

Cerebral capillary ECs differ from other ECs in that they have a larger number of tight junctions between neighboring cells, fewer cytoplasmic vesicles and higher amounts of mitochondria (Oldenhorf and Brown, 1975). The tight junctions limit paracellular movement and divide the membranes of ECs into two distinct sides with different membrane composition (Van Meer and Simons, 1986; Tewes and Galla, 2001; Hawkins et al., 2006, 2013). In addition, transcellular flux is also restricted, as these ECs show extremely low transcytotic activity (Coomber and Stewart, 1985). Thus, molecules must pass two sheaths of membrane to enter or leave the brain so that transporters located on each side of the cell membrane play a pivotal role in controlling this movement. In this regard, the higher amount of mitochondria in these cells contributes toward generating more ATP molecules to drive the ion gradients which are critical for transport functions. All in all, the combination of physical barrier properties, together with specific transporters to deliver required nutrients, allows the ECs to tightly regulate CNS homeostasis. Several approaches have been undertaken to elucidate the different transporters, their expression and specific location. For example, isolation of brain microvessels and further genomic and proteomic analysis may permit the identification of the cellular location of proteins expressed within the neuro-vascular unit [for a review, see Pardridge (2020)].

Ultimately, transport across the BBB is an important step in the regulation of nutrient and metabolite movement between blood and the brain. Endothelial cells show a complex network of specific transporters present in one or both cell membranes (luminal and abluminal) to regulate this flow. It is the combined characteristics of both membranes that determine which molecules traverse the BBB and how fast (Valdovinos-Flores and Gonsebatt, 2012). In fact, there are 244 genes belonging to the SLC (solute carrier) superfamilies that are expressed in brain microvessels (Geier et al., 2013), including *slc2a*1(glucose), *slc16a1* (lactate, pyruvate), *slc7a1* (cationic amino acids), and *slc7a5* (neutral amino acids, L-DOPA) (Zlokovic, 2008; Daneman and Prat, 2015). This paper gives an overview of these SLC transporters specific for AA transport, precisely pinpointing their polarized location, their substrates and their direction of transport.

## Amino Acid Transporters

**FIGURE 1:**
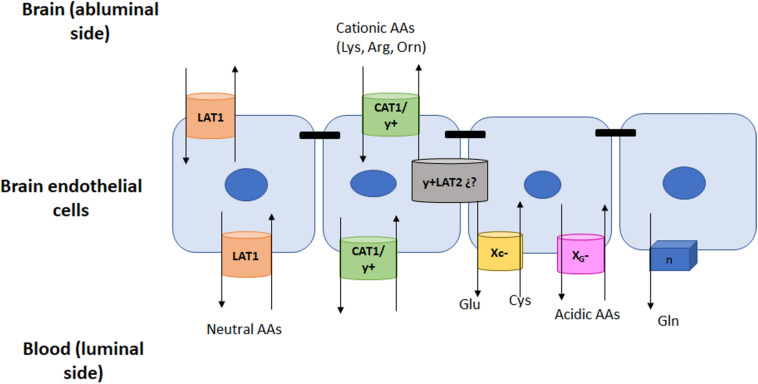
AAs Facilitative Na^+^-independent transporters in the BBB. Amino acids are transported by systems L and y^+^ from blood to ECs and then into the brain. These two systems are located at both sides of the cell membrane. However other systems will also be present but exclusively at the luminal side of the BBB. The location of these transporters and their main substrates is depicted in the figure.

**FIGURE 2:**
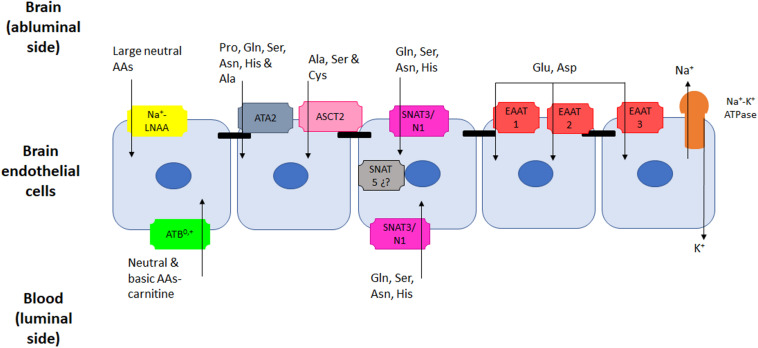
AAs active transporters present in endothelial cells of the BBB. At least five different transport systems that are Na^+^-dependent have been described in the BBB. Of those, only system ATB^0,+^ that transports neutral and basic AAs and one member of system N (SNAT3 for basic AAs) have been detected in the luminal membrane. The other systems for large neutral AAs (Na^+^-LNAA), alanine (System A), small neutral (System ASC), basic (System N) and acidic (EAATs) are all located in the abluminal membrane, capable of pumping AAs out of the brain against a concentration gradient.

The concentration of AAs in brain extracellular fluid and cerebral spinal fluid is at least tenfold lower than plasma concentrations. The only exception to this being glutamine, the concentration of which is similar at both sides of the BBB (O’Kane and Hawkins, 2003; Hawkins et al., 2013; Nalęcz, 2017). This gradient, as well as AA transport across the BBB is tightly regulated by AA transporters present in both membranes of ECs; in fact, ten AA transport systems with different membrane distribution have been reported (Valdovinos-Flores and Gonsebatt, 2012) (for localization of these transporters, see Figures 1 and 2).

In a broad sense, these transporters can be classified according to their presence in the luminal or abluminal membranes, or in both. Depending on the transport mechanisms used, these AA transporters can be antiporters, which exchange some AAs for others across the membrane by means of facilitative transport. On the other hand, symporters co-transport AAs together with ions along the electrochemical gradient of the ions and are active transporters (Taslimifar et al., 2018). Five active transporters that require Na^+^ are present in the abluminal membrane and are responsible for the AA efflux from the brain into the ECs and from there to the blood. Furthermore, facilitative-carriers, which are sodium-independent, are located either in the luminal side or in both luminal and abluminal membranes (see Tables 1 and 2 for names, substrates and location of each transporter).

**TABLE 1:** Overview of the nomenclature of facilitative amino acid transporters on the blood-brain barrier.

System	Hugo name	Other names	Substrates	Polarity
System L	**SLC7A5** (solute carrier family 7 member 5)	LAT1 (amino acid transporter light chain, L system)	Large neutral AAs (Phe, Trp, Leu, Ile, Met, His, Tyr, Val and Thr)	Luminal & Abluminal
System CAT or y^+^	**SLC7A1** (solute carrier family 7 member 1)	CAT1 (cationic amino acid transporter, y^+^ system)	Cationic AA (Lys, Arg, Orn)	Luminal & Abluminal
	**SLC7A2** (solute carrier family 7 member 2)	CAT2 (cationic amino acid transporter, y^+^ system)	Cationic AA (Lys, Arg, Orn)	Luminal & Abluminal
	**SLC7A3** (solute carrier family 7 member 2)	CAT3 (cationic amino acid transporter, y^+^ system)	Cationic AA (Lys, Arg, Orn)	Luminal & Abluminal
System BAT (y^+^LAT2)	**SLC7A6** (solute carrier family 7 member 6)	y^+^ LAT-2 (amino acid transporter light chain, y^+^ L system)	Arg/Gln exchange	Unknown
System Xc^–^	**SLC7A11** (solute carrier family 7 member 11)	xCT (anionic amino acid transporter light chain, xc^–^ system)	Cys2/Glu exchange	Luminal

*The nomenclature used here is the one adopted by the HUGO Gene Nomenclature Committee.*

**TABLE 2:** Overview of the nomenclature of Na^+^-dependent active amino acid transporters on the blood-brain barrier.

System	Hugo name	Other names	Substrates	Polarity
System BAT (ATB^0,+^)	**SLC6A14** (solute carrier family 6 member 14)	ATB^0,+^	Neutral and basic AA	Luminal
System A	**SLC38A2** (solute carrier family 38 member 2)	ATA2, SAT2, SNAT2	Small AA (Ala, Pro, His, Ser, Asn)	Abluminal
System ASC	**SLC1A4** (solute carrier family 1 member 4)	ASCT1	Ala, Ser, Cys	Abluminal (Embryonic development)
	**SLC1A5** (solute carrier family 1 member 5)	AAAT; ASCT2	Ala, Ser, Cys	Abluminal
System N	**SLC38A3** (solute carrier family 38 member 3)	SN1; SNAT3	Nitrogen-rich AA: Gln, His, Asn	Luminal & Abluminal
	**SLC38A5** (solute carrier family 38 member 5)	SN2; SNAT5	Nitrogen-rich AA: Gln, His, Asn	Unknown
EAATs	**SLC1A3** (solute carrier family 1 member 3)	EAAT1; GLAST solute carrier family 1 (glial high affinity glutamate transporter), member 3	Glu and Asp	Luminal
	**SLC1A2** (solute carrier family 1 member 2)	EAAT2; GLT-1 solute carrier family 1 (glial high affinity glutamate transporter), member 2	Glu and Asp	Luminal
	**SLC1A1** (solute carrier family 1 member 1)	EAAT3; EAAC1 solute carrier family 1 (neuronal/epithelial high affinity glutamate transporter, system Xag), member 1	Glu and Asp	Luminal

*The nomenclature used here is the one adopted by the HUGO Gene Nomenclature Committee.*

## Facilitative Transport Across the BBB: Transporters Present in Both Membranes

Two sodium independent transport systems, LAT1 or L, and y^+^, mediate the facilitated exchange of large neutral AAs (both systems) and basic AAs (y^+^) in both luminal and abluminal membranes of ECs. They play a key role in delivering dietary essential neutral and basic AAs that cannot be synthesized within the brain (Stoll et al., 1993; Sanchez del Pino et al., 1995; Figure 1).

### Large Neutral Amino Acid Transporters

Neutral AAs are transported across the plasma membrane by proteins that belong to the solute carrier families SLC1, SCL7, SLC38 and SLC43 (Kanai and Hediger, 2004). Of these SLC families, only SLC7 and SLC43 are Na^+^-independent and belong to system L amino acid transporter (system LAT), while SLC38 (Systems A and N) and SLC1 (System ASC) are Na^+^-dependent transporters and will be discussed further on. Early studies *in vivo* on transport across the BBB revealed that neutral AA moved from blood to brain by facilitative Na^+^-independent transport by means of a transmembrane transporter known as LAT1 (from system L amino acid transporter) (Boado et al., 1999; Hawkins et al., 2013). LAT1 is encoded by the *slc7a5* gene and forms a heterodimeric complex with the glycoprotein 4F2hc (CD98, SLC3A2) associated by a disulfide bridge. Recently, it has been shown that LAT1 is in charge of AA transport (Napolitano et al., 2015), whilst 4F2hc heavy subunit acts as a molecular chaperone that directs LAT1 to its definitive localization in the cellular membrane (Nakamura et al., 1999).

This transporter is highly expressed in the ECs of the BBB. Regarding polarity, LAT1 is present in both membranes in a 2:1 ratio (luminal vs. abluminal) (Sanchez del Pino et al., 1995; Duelli et al., 2000). A computational study supports the hypothesis that there is an asymmetry in either bi-directional kinetics and/or expression of LAT1 in brain ECs which in turn results in a functional polarity for these large neutral AAs transported by LAT1 (Taslimifar et al., 2018).

At the BBB, LAT1 is stereospecific (L > D), catalyzes an exchange (in 1:1 stoichiometry) of two AAs and is inhibited by BCH [2-aminobicyclo-(2,2,1) heptane-2-carboxylic acid], an amino acid-related compound that has been used as a selective inhibitor of the system L amino acid transporters. It preferentially transports branched AAs, and their substrates including Phe, Trp, Leu, Ile, Met, His, Tyr, Val and Thr, most of which are essential AA. Gln has also been described as a substrate for this transporter; however, Gln transport is not completely inhibited by BCH, suggesting that Gln has an additional Na^+^-independent transport system in the BBB, most likely system n (Lee et al., 1998). It has also been demonstrated that LAT1 shows different affinities on extracellular vs. intracellular sides; indeed, it has higher affinity for intracellular amino acids whose concentration controls the transport rate (Meier et al., 2002). According to this, L-leucine, L-isoleucine and L-methionine are better substrates for cellular efflux than influx to the cell (Fotiadis et al., 2013).

LAT1 imports large and neutral amino acids in exchange for intracellular AAs (e.g,. glutamine), therefore it seems to be the most important path for neutral essential AAs to enter the brain (Fotiadis et al., 2013; Singh and Ecker, 2018). The homeostasis of these neutral AAs (which are asymmetrically distributed in plasma and brain) is essential, since some of them play a crucial role as precursors for neurotransmitters, such as dopamine, serotonin and histamine.

### Cationic Amino Acid Transporter: System y^+^

The concentration of cationic amino acids (CAA; lysine, arginine, and ornithine) in ECF is 10–30% that of plasma although their transport across the BBB is less well known. Two families of proteins have been described as CAA transporters: CAT or cationic amino acid transporters system y^+^ (SLC7A1-4), which is selective for basic AA (index “ + ”); and BAT or broad substrate amino acid transport systems (that include B^0,+^, b^0,+^, and y^+^LAT2), which also transport neutral amino acids (index “0”) (Deves and Boyd, 1998; Hawkins et al., 2013). Transporter system B^0,+^ (or ATB^0,+^) is a symporter with 2Na^+^ and 1Cl^–^, therefore it will be discussed in the Na^+^-dependent transporters section. Indeed, it is the only Na^+^-dependent carrier that transports CAA. On the other hand, the presence of the Na^+^-independent systems b^0,+^ and y^+^LAT2 in either membrane of the BBB remains to be elucidated (O’Kane et al., 2006). Moreover, the Arg/Gln exchanger y^+^LAT2 (SLC7A6) appears to be expressed in the cerebral capillary endothelial cells in human *in vitro* BBB models (Carl et al., 2010), or even after ammonia treatment in cultured rat ECs (Skowronska et al., 2012). However, there is no information concerning its specific localization in the brain endothelium *in vivo*.

Furthermore, system y^+^ (CAT1, CAT2B and CAT3) seems to be the primary CAA transporter of the BBB; it has been known to be present in the luminal side since the early 1990s (Smith, 1991). More recent studies with enriched luminal and abluminal vesicles from the bovine BBB demonstrated that transporters from system y + also exist on the abluminal membrane (O’Kane et al., 2006). In fact, their activity is greater on the abluminal membrane and they are voltage-sensitive. Although CAT1 is the best characterized cationic AA transporter, other members have also been studied in the brain. CAT2 is expressed in the brain, but does not appear to be enriched at the BBB, since mRNA levels are <10% of that of CAT1 (Smith, 2000). CAT3 is highly expressed in the brain and readily discriminates between L-lysine and L-arginine (difference of approximately 2-fold).

CAT1 (SLC7A1) has a higher affinity for Arg compared to the other CAA and is responsible for blood-to-brain transport of Arg across the BBBs (Stoll et al., 1993). Since CAT1 forms a caveolar complex with endothelial nitric oxide synthase (eNOS) (McDonald et al., 1997), CAT1 is probably the most important player in providing Arg for NO synthesis in endothelial cells (O’Kane et al., 2006). Despite being primarily a CAA transporter, system y^+^ also exhibits weak interactions with neutral AA in the presence of sodium. In the BBB, this system may transport several NAA, both essential (Phe, Thr, His, Val, and Met) and non-essential (Ser, Gln, Ala, and Gly) although with much less affinity than system LAT1 (Hawkins et al., 2013).

## Facilitative Transport Exclusively Found on the Luminal Side

There are at least three different Na^+^-independent facilitative carriers found at the luminal membranes of ECs. These transporters that do not require energy for the movement of their substrates are not as abundant as the previously mentioned LAT and y^+^ systems. Indeed, they do not account for the movement of a large group of AAs but for specific molecules instead. Accordingly, these carriers regulate the efflux from the brain to plasma of glutamine and acidic AAs, mainly glutamate which can be neurotoxic at high levels. On the other hand, cysteine needs to be imported from plasma into the brain in order to synthetize glutathione (GSH), and this import is mediated by system X_C_^–^ (Figure 1).

### Glutamine Transporter: System n

L-glutamine (Gln) is the most abundant AA in plasma and cerebrospinal fluid and is a precursor for the main central nervous system excitatory (L-glutamate) and inhibitory [γ-aminobutyric acid (GABA) neurotransmitters]. Multiple transporters have been identified that are capable of glutamine transport, at both surfaces of the BBB. These include Na^+^-dependent transporters that belong to systems N, A, ASC, B^0,+^, and y^+^LAT2, and Na^+^-independent transporters from systems L, b^0,+^ and n.

Despite its potential importance, the mechanisms of Gln transport across the BBB and the polarity of these transporters has not been fully elucidated. What is certain is that Gln moves from the plasma across the BBB by a facilitated process. Initially, it was assumed that Gln was transported across the BBB on the neutral amino acid transporter designated system L (or LAT1), and in fact it does. However, this is not the only system capable of transporting Gln. Indeed, Lee et al. (1998) demonstrated that Gln transport into the brain was not inhibited by BCH, a specific inhibitor of LAT1, and did not demonstrate trans-stimulation, suggesting that Gln facilitative transport across the luminal membrane of the BBB occurred by a different system, which was coined *system n* due to its similarities to system n described in hepatic plasma membrane vesicles.

Nevertheless, other authors point to Systems L (facilitative-transporter) and N (active transport) as having a predominant role in Gln homeostasis, due to abundant expression and endothelium membrane localization of LAT1 and SNAT3 (SCL38A3) (Dolgodilina et al., 2016). In agreement with this hypothesis, the active-transporter system N exporting Gln out of the brain coupled with Na^+^ was thought to be restricted to the abluminal membrane, but the presence of some members of this system on both sides has been detected in murine and rat models (Nalęcz, 2017).

### Glutamate Transport: Systems X_G_^–^ and X_C_^–^

Studies using isolated membranes from the BBB revealed that facilitative carriers for glutamate (Glu) only existed on the luminal side, while sodium-dependent active co-transporters were confined to the abluminal membrane (Lee et al., 1998). Initially, it was tacitly assumed that this Na^+^-independent transporter was a path for Glu entry into the brain. Several facts contradicted this assumption: (1) Glutamate is a non-essential AA, but it is the most abundant AA in brain, (2) Large doses of Glu within the brain extracellular fluid (physiological range is 0.5–2 mM) induce overexcitation in neurons and thus, this AA is neurotoxic, so its concentration must be tightly controlled, (3) Glu does not enter the brain in appreciable amounts, except in the circumventricular organs and finally, (4) Glu is synthetized in the brain in large quantities. All this evidence indicated that active transporter on the abluminal side triggers the release of Glu from cerebrospinal fluid (CSF) into ECs and, concomitantly, facilitative carriers on the luminal membrane mediate Glu efflux from ECs to plasma (Hawkins and Viña, 2016).

Regarding the specific facilitative transporters for Glu on the luminal side, there are two plausible candidates. The removal of Glu from ECs to plasma can be mediated by an L-glutamate/L-cystine (Cys_2_) exchanger, called system X_C_^–^. It consists of a catalytic subunit xCT (SLC7A11) that heterodimerizes with a heavy chain 4F2hc (SLC3A2), similarly to LAT1. High mRNA levels of xCT and protein expression are found in border areas of the brain, between the brain and the periphery, such as ECs, choroid plexus, and ependymal cells (Sato et al., 2002; Burdo et al., 2006). System X_C_^–^ is responsible for Cys_2_ influx in the luminal membrane of ECs in exchange for internal glutamate; it is a Cys_2_ transporter that uses the transmembrane gradient of glutamate as the driving force. In agreement with this, it has been demonstrated that uptake of cystine causes glutamate release and that extracellular glutamate inhibits uptake of cystine. Once Cys_2_ is incorporated into the ECs it is rapidly reduced to Cys, which will either be taken for protein or GSH synthesis within the cells or transported into the CNS through the abluminal membrane by system L.

A second Na^+^-independent system found in the luminal membrane was described in 1996 (Benrabh and Lefauconnier, 1996). Their experiments demonstrated that cysteine did not compete with Glu for uptake, showing that the transport system located at the luminal side was not X_C_^–^. Moreover, Asp which is not a substrate for Xc^–^ system, inhibited Glu transport, suggesting that it was X_G_^–^ the transporter they could detect in their assays. The exact protein that mediates this uptake has yet to be identified.

Both L-glutamate and L-aspartate are “dietary non-essential” AAs, which can be synthesized readily in the brain, so they show much lower rates of uptake into the brain at the BBB (Benrabh and Lefauconnier, 1996; Smith, 2000); instead brain supply is driven more by intracerebral synthesis and breakdown. Furthermore, Hawkins et al. (1995) reported that the brain uptake for Glu is 5- to 10-fold less than that of most of the large neutral and basic amino acids carried by systems L and y^+^.

## Active Transport Across the BBB

As previously mentioned, the concentration of all naturally occurring AAs in CSF and brain extracellular fluid is 10-20 times lower than in plasma. The only exception is glutamine, whose concentration is similar in CSF and plasma. This gradient on both sides of the BBB is strictly controlled by the different systems of AA transporters. Indeed, AAs leaving the brain toward the circulation are transported against a concentration gradient, so energy is needed. Consequently, active transporters located in the abluminal side of the BBB export AAs from the brain into the endothelial cell, to keep the lower concentration found in the extracellular fluid. These active transporters couple AA exit with Na^+^ transport. To maintain the sodium gradient required, a Na^+^-pump is also present at the abluminal membrane of ECs.

Five Na^+^-dependent systems have been reported in the abluminal side, and more recently some of these systems have also been described on luminal membranes. These systems are: (1) a system for large neutral AA (LNAA); (2) system A (Ala preferring), which was characterized first and transports other small non-essential neutral AAs; (3) system ASC, whose name derived from the main substrates Ala, Ser, and Cys; (4) basic AA transport (Gln, Asn, and His) which is system N; and finally (5) a system for acidic AA, the family of excitatory amino acids transporters (EAATs) that carry preferentially Asp and Glu. (See Table 2 for more details on name, substrates and location of these active transporters) (Figure 2).

These active transporters were thought to be located only in abluminal membranes, and this would reinforce the idea that these carriers remove AAs from the brain benefiting from the Na^+^-gradient that exists between CSF and ECs (Hawkins et al., 2013). However, more recent publications have shown the presence of Na^+^-coupled transport at the luminal membranes; this is the case of a carrier from the BAT system (broad substrate amino acid transport) that is B^0,+^ (or ATB^0,+^) (Michalec et al., 2014) or even for SNAT3 (a member of system N), that has been located in the mouse capillary luminal and abluminal membranes (Ruderisch et al., 2011).

### Transport of Large Neutral AAs: System Na^+^-LNAA

Initially a Na^+^-dependent transporter for Phe that was inhibited by BCH was described in the BBB (Sanchez del Pino et al., 1995). Since system ATB^0,+^ (SLC6A14) was known as a Na^+^-dependent transporter that could be inhibited by BCH [2-aminobicyclo-(2,2,1) heptane-2-carboxylic acid], the authors thought that system ATB^0,+^ was likely to be responsible for Phe transport. However, this hypothesis has never been confirmed, and more recent publications have described the presence of this transporter in the luminal membrane of rat brain slices (Michalec et al., 2014). ATB^0,+^ is a plasma membrane transporter, specific for neutral and basic AAs, with the highest affinity for isoleucine and leucine. It transports its substrates in a symport with 2 Na^+^ and 1 Cl^–^, coupled to the membrane potential. *In vitro* experiments with cultured bovine brain capillary ECs showed mRNA levels of this transporter (Berezowski et al., 2004); but other authors could not detect it in freshly isolated BBB mouse endothelial cells (Lyck et al., 2009), therefore more research is needed. Apart from neutral AAs, ATB^0,+^ can also transport carnitine, not synthesized in the brain, which is an important precursor of some neurotransmitters (Michalec et al., 2014).

Another system, also sensitive to BCH inhibition when transporting Phe, has been described on the abluminal side of cow brain capillaries (O’Kane and Hawkins, 2003). It is known as system Na^+^-LNAA (from Na-dependent large neutral amino acids); it has a high-affinity for Leu and is inhibited by other NAAs including Gln, His, Met, Phe, Ser, Thr, and Tyr. The spectrum of substrates is similar to that of system LAT1 that transports essential NAAs with a concentration gradient. It seems that Na^+^-LNAA in the abluminal membrane removes essential NAAs from the brain, and thus, provides a mechanism for controlling LNAA flux and concentration in CSF (Hawkins et al., 2013). Nevertheless, the molecular identity of system Na^+^-LNAA remains to be elucidated. According to Nalec KA (Nalęcz, 2017), a possible candidate could be SBAT1 (SLC6A15), present in the brain and capable of transporting LNAA (Takanaga et al., 2005). Its transport is Na-and pH-dependent, however it does not require Cl^–^ ions. Despite its presence in the brain, little is known about its expression or localization in the BBB. Moreover, the substrate specificities of Na^+^-LNAA and SBAT1 are not the same (Nalęcz, 2017), so more research needs to be undertaken to decipher the gene which encodes for this transporter.

### Transport of Small Non-essential Neutral AAs: System A

Alanine, serine and cysteine can be transported by the sodium-dependent system A (named A for its preference for Ala as a substrate). This system was found in the abluminal membrane of bovine BBB (Sánchez del Pino et al., 1992) and it accepts MeAIB [N-(methylamino)isobutyric acid] as a unique substrate, which distinguishes it from other active carriers. It is a voltage-sensitive transporter; three positive charges are translocated per MeAIB molecule (Hawkins et al., 2013). System A mediates the efflux from the brain of small AAs, such as Pro, Gln, Ser, Asn, His, and Ala, helping to maintain the low concentration of these AAs within the CSF. Of the different members of this family, only ATA2 (SLC38A2) has been demonstrated to be expressed in the rat BBB and its expression increases under hypotonic conditions (Takanaga et al., 2002; Nalęcz, 2017). Indeed, this system accounts for approximately 20% of abluminal Na-dependent Gln transport activity, whereas system N mediates the majority of Gln transport across the abluminal membrane (Lee et al., 1998).

### Transport of Large and Small Neutral AAs: System ASC

This is a Na^+^-dependent system located exclusively on the luminal membranes of the BBB, whose name “ASC” was derived from the three main substrates Ala, Ser, and Cys, although Gly and several essential AAs are also putative substrates, including Met, Val, Leu, Iso, and Thr (Hawkins et al., 2013). The presence of this transport system was confirmed by inhibiting system A with MeAIB; ASC activity was not inhibited, and Ala could be transported by this second system (Tayarani et al., 1987). Kinetic studies also revealed that ASC activity is independent of the transmembrane potential (Hawkins et al., 2013).

At least two members of this ASC system have been described in different BBB models. On the one hand, ASCT1 (SLC1A4) was observed in brain ECs during embryonic and neonatal development, but not in adult brains (Sakai et al., 2003). Furthermore, ASCT2 (SLC1A5) is expressed in higher levels than ASCT1 in the mouse BBB and it was localized at the abluminal side by immunohistochemical analysis (Tetsuka et al., 2003; Gliddon et al., 2009).

### Transport of Nitrogen-Rich AAs: System N

System N accounts for its preference in AA substrates that are nitrogen-rich, such as His, Gln and Asn, although Ser can also be transported through this system (Hawkins et al., 2013). This transporter exports AAs in a symport with Na^+^ and antiport with H^+^ and is voltage-independent. It was first described in the rat BBB at the luminal membrane and it was pH sensitive and moderately tolerant of Li^+^ substitution for Na^+^ (Ennis et al., 1998). However, more recently, the presence of SNAT3 (SLC38A3) from the system N amino acid transporter was detected in both sides of the BBB in mouse ECs (Ruderisch et al., 2011), while transcripts of SNAT5 (SLC38A5) have also been detected in mouse BBB (Dahlin et al., 2009).

### Transport of Acidic AAs: EAATs Transporters

Acidic amino acids Glu and Asp are transported from brain to blood; at the abluminal membrane this efflux is mediated by excitatory amino acid transporters (EAAT), encoded by genes from an SLC1 family. These transporters are voltage-dependent and export Glu and Asp together with 3Na^+^ and 1H^+^ in a counter-transport of 1K^+^ (Nalec 2016). Currently, five members of the EAAT family have been identified in the plasma membranes of astrocytes and neurons (Miralles et al., 2001; Fahlke et al., 2016) as well as in the BBB (O’Kane et al., 1999). Three EAAT members (EAAT1-3) have been detected in the abluminal membrane of the BBB, in different in vivo and in vitro models (O’Kane et al., 1999; Cederberg et al., 2014). cDNAs for these three transporters were isolated from the ECs of cerebral capillaries and experiments with luminal and abluminal membranes demonstrated that they were present exclusively in the abluminal membranes (O’Kane et al., 1999; Cohen-Kashi-Malina et al., 2012). Regarding other members of this family, a transcript for EAAT4 was detected in brain ECs (Lehre et al., 1995; Enerson and Drewes, 2006).

Experiments with specific inhibitors showed that the activity ratio of the three transporters EAAT1: EAAT2: EAAT3 found at the abluminal membrane was 1:3:6, respectively. This family of EAATs is the most powerful of all Na^+^-dependent transporters of the abluminal side. It was proposed that these transporters play a key role maintaining the glutamate gradient between brain cells and extracellular fluid, since they are the most important players because they catalyze Glu efflux from the brain.

## Transport of Taurine Across the BBB

Taurine (2-amino-ethanesulfonic acid) is a sulfur-containing β-amino acid which is quite abundant in the brain. It is synthetized in the liver and enters and leaves the brain through different transporters. Inside the brain it can be an osmoregulator and a neurotransmitter (Wu and Prentice, 2010). It has been demonstrated that abluminal membranes had both Na^+^-dependent taurine transport as well as facilitative transport while luminal membranes only had facilitative transport (Rasgado-Flores et al., 2012). However, the system for facilitative transport has yet to be identified.

Regarding the active transport system, presence of the taurine transporter TAUT (SLC6A6) has been shown in rat brain ECs (Kang et al., 2002). This transporter is Na^+^ and Cl^–^-dependent, it is also voltage-dependent and sensitive to hypertonic conditions (Kang et al., 2002; Rasgado-Flores et al., 2012; Hawkins et al., 2013). It can be regulated at a transcriptional level by TNFa which also diminishes the efflux of taurine through the BBB *in vivo* (Lee and Kang, 2004). In 2012, Rasgado-Flores et al. (2012) demonstrated in experiments with isolated vesicles from plasma membrane that this active transporter was only located in the abluminal membrane of the BBB. This indicates that taurine can be depleted from the brain even against a concentration gradient across the abluminal membrane.

## Concluding Remarks: Organization of the Different Transport Systems

The BBB participates in the control of AA concentrations in the brain, not only for their transport from plasma into the CSF but also for their active removal from the brain through the Na+-dependent transporters located in the abluminal membrane. Systems LAT1 and y+, by being at both sides of the EC plasma membrane, play a central role providing the brain with all essential AAs required. However, there is always more than one transporter capable of moving one specific AA from one side to the other; indeed, there is a substrate overlap among the facilitative systems (Table 1), as well as within the active transporters (Table 2).

The abluminal efflux of AAs from the brain into the ECs and then to the blood takes place against a concentration gradient (∼10-fold higher concentration of AAs in plasma) and thus requires energy. To fulfill this need, there are several Na^+^-dependent systems located in the abluminal side and a Na^+^/K^+^ ATPase that creates the electrochemical gradient of Na^+^ toward the ECs, allowing the transport of AAs out of the brain (Hawkins et al., 2013). Moreover, these active transporters, located at the abluminal membrane, provide a mechanism for removing essential and non-essential AAs and also other excitatory AAs that can be toxic at higher concentrations. As in the case of facilitative carriers, the majority of AAs can be exported from the brain by more than one transporter, ensuring their removal from the brain and therefore maintaining an optimal concentration within this tissue (Table 2).

The complexity and coupling of all these transport systems relies on the fact that not only ion gradients or voltage gradients regulate transport across the BBB; other molecules might also have a role. This is the case for oxoproline (pyroglutamate), an intermediate molecule of the γ-glutamyl cycle. It has been described that the BBB exhibits a high γ-glutamyl transpeptidase activity (Orlowski et al., 1974) and that this enzyme is an integral protein of the luminal membrane of the BBB (Lee et al., 1996). A role for γ-glutamyl transpeptidase in AA transport has already been suggested by the group of Meister and others, in several tissues other than the BBB (Orlowski and Meister, 1970; Viña et al., 1981, 1989, 1990). In fact, intracellular oxoproline stimulates the Na^+^-dependent systems, especially system A located at the abluminal membrane (Lee et al., 1996). This is of particular interest to control the concentration in the brain of small non-essential AAs (which are the main substrates for system A). Thus, the regulation of AA transport by oxoproline may serve to modulate availability of AAs that act as neurotransmitters or their precursors (Hawkins and Viña, 2016).

Although it has been generally assumed that Na^+^-dependent transporters were confined to the abluminal membrane, at least two active transporters have been described in the luminal side, ATB^0,+^ and SNAT3 (system N). ATB^0,+^ belongs to the broad spectrum of AA transporters and its presence at the luminal side could be explained as a gate-keeper, to ensure AA delivery into the brain under certain pathological conditions. It should also be considered that both systems, ATB^0,+^ and SNAT3, transport Gln which is the only AA whose concentration is in the same range at both sides of the BBB. Although it is accepted that Gln is transported from the brain to blood, the presence of these transporters at the luminal side could also ensure appropriate levels of this AA when needed.

Finally, it has to be taken into account that some of these AAs are neurotransmitters or precursors and their movement through the BBB is crucial for CNS function. Tyr is a precursor of catecholamines and Trp is a serotonin precursor. These AAs can enter the brain ECs guided by a concentration gradient and there are several systems that allow this entrance (Systems LAT1, Na^+^-LNAA as well as ATB^0,+^). On the other hand, some AAs have to be taken out of the brain, which is the case of Glu. An excess of Glu is neurotoxic since it induces hyperexcitation. It has to be removed from the brain extracellular fluid through EAATs transporters (the most powerful of the active transporters) present in astrocytes (EAAT1-3), neurons (EAAT3) and at the abluminal side of the BBB (EAAT1-3) and through systems Xc^–^ and X_G_^–^ in the luminal membrane. Another AA related to Glu is Gln, which is pumped from brain extracellular fluid into ECs mainly by two active systems, systems A and N (which account for 80% and 20% of Gln release, respectively). Once inside ECs it can also reach circulation by means of facilitative carriers or even be converted into Glu in a reaction catalyzed by glutaminase. In any case, these Glu molecules will traverse the luminal membrane and reach plasma. The BBB restricts the entrance of Glu and Gln; it actively exports these two AAs to the blood instead, protecting the brain from neurotoxicity generated by accumulation of Glu.

Glutamate exerts as an excitatory neurotransmitter by activating a range of neuronal receptors, among them N-methyl-D-aspartate receptors (NMDARs); and these receptors are responsible for many physiological and pathophysiological roles of Glu within the brain. NMDAR have been found in ECs forming the BBB. Indeed, when these receptors are activated by uncontrolled levels of Glu they may disrupt BBB integrity, altering the expression of efflux transporters or reducing the number of tight junctions within the ECs (Hogan-Cann and Anderson, 2016). Moreover, endothelial NMDARs may also affect transport across the BBB in response to Glu levels. These receptors might influence vascular diameter, mediating vasodilation and rising blood flow in brain endothelial cells which in turn increases substrate availability in the BBB.

All in all, the organization of these transporters within the BBB maintains and strictly controls the AA concentrations in brain extracellular fluid. The unequal distribution of those systems between the luminal and abluminal membranes serves to catalyze uptake of specific AAs by the brain and removal of some others. The fact that more than one transporter may carry the same substrate ensures supply and excretion of AAs for proper functioning of the brain and brain homeostasis.

## Author Contributions

The author confirms being the sole contributor of this work and has approved it for publication.

## Conflict of Interest

The author declares that the research was conducted in the absence of any commercial or financial relationships that could be construed as a potential conflict of interest.
